# Immunohistochemical Expression of *Cyclin D1* and *Ki-67* in Primary and Metastatic Oral Squamous Cell Carcinoma

**DOI:** 10.31557/APJCP.2020.21.1.37

**Published:** 2020

**Authors:** Maryam Nazar, Iram Naz, Muhammad Khurram Mahmood, Shoaib Naiyer Hashmi

**Affiliations:** 1 *Department of Oral Pathology, Margalla Institute of Health Science, *; 4 *Department of Histopathology, Armed Forces Institute of Pathology, Rawalpindi, *; 2 *Department of Oral Pathology, Islamabad Medical and Dental College, *; 3 *Department of Pharmacology and Therapeutics, HBS Medical and Dental College, Islamabad, Pakistan. *

**Keywords:** Squamous cell carcinoma, Cyclin D1, Ki-67, metastasis

## Abstract

**Objective:**

The aim of current study was to investigate the expression of *Cyclin D1* and *Ki-67 *in primary and metastatic oral squamous cell carcinoma (OSCC) and their different histological grades.

**Methods:**

Paraffin embedded 30 oral squamous cell carcinoma (15 each of primary and cervical lymph node metastatic OSCC) were included in the study. *Cyclin D1* and *Ki 67* expressions were evaluated by immunohistochemistry and compared in primary and lymph node metastasis of OSCC and their histological grades. The data was analyzed using SPSS software.

**Results:**

The mean age of patients with primary OSCC was 53.47 ±16.67 years and 61.47 ±11.94 years in patients with metastasis. Males were comparatively affected more than females with tongue as the most common site involved in both primary and metastatic tumours. The mean size of primary and metastatic tumour biopsies were 1.16 mm and 3.93 mm respectively. Comparison of the expression of* Cyclin D1* in these primary and metastatic OSCC revealed a statistically significant difference (p = 0.003) whereas it was insignificant for* Ki-67* (p = 0.715).

**Conclusion:**

*Cyclin D1* can be a useful marker in predicting aggressive or metastatic behaviour of OSCC on premier biopsies.

## Introduction

Oral cancers are claimed to account 2-4 % of all cancers worldwide and amongst all Oral Squamous Cell Carcinoma (OSCC) is the most frequently occurring. It constitutes over 90% of malignancies found in the mouth and oropharynx; considered malignant due to its infiltrative capacity and early tendency to metastasis (Yakob, 2014; Acero-Mondragón et al., 2016). Tobacco smoking, betel quid chewing and alcohol consumption are some of the associated risk factors resulting in its development (Mishra et al., 2015).

Although the number of molecular-based assays has increased in recent years but still histopathology remains the gold standard for most diagnostic and therapeutic decisions. Immunohistochemistry which is available globally complements histopathological analysis by detecting gene expression at the protein level (Oliveira and Ribeiro-Silva, 2011). Similarly advances in understanding of the molecular mechanisms underlying OSCC have resulted in an increasing number of biomarkers that can be used to predict its behaviour. *Cyclin D1* is a vital proto oncogene that has a major role in cell cycle regulations through G1 to S phase progression. It is important for the development and progression of several cancer types, including oral epithelial cancer. Abnormal *Cyclin D1 *expression, either by rearrangement, amplification or transcriptional up-regulation contributes to the loss of normal cell cycle control correlated to an increased risk of tumorigenesis (Zhao et al, 2014). Over expression of CyclinD1 is reported to be associated with recurrence and shortened overall survival in curable cases of squamous cell carcinoma of head and neck (SCCHN) (Shiang-Fu et al, 2012). 

*Ki-67* is another nuclear protein, a cellular marker for proliferation expressed in the G-2 and M-phases of actively dividing cells. It correlates with the presence and severity of epithelial dysplasia and provides significant information about the degree of aggressiveness and prognosis of OSCC. It is also an excellent marker to determine the growth fraction of a given cell population. The fraction of *Ki-67*-positive tumour cells is often correlated with the clinical course of cancer. Immunohistochemical evaluation for *Ki-67* antigen analysis has been the most prevalent method (Inwald et al., 2013). It was therefore hypothesized that *Cyclin D1* and *Ki-67* will have more association with metastatic and poorly differentiated tumours.

Hence, the main objective of the current study was to investigate the expression of these immune-histochemical markers in primary and cervical lymph node metastatic OSCC and their various histopathologic grades.

## Materials and Methods

*Patients/ Specimens*

This retrospective study utilised formalin-fixed, paraffin-embedded tumour samples of 30 patients histologically diagnosed with primary and cervical lymph node metastatic OSCC (15 of each type) sourced from the archival of Histopathology Department of Armed Forces Institute of Pathology (AFIP), Rawalpindi, Pakistan. The study was approved initially by Ethics Committee of AFIP and subsequently from the 83rd meeting of Advanced Studies and Research Board of University of Health Sciences (Lahore), Pakistan.

The demographic details (age, gender, site of the lesion) were recorded from the clinical histories/ data presented with each biopsy. Both previously and freshly diagnosed primary OSCC patients treated with neck dissection belonging to either sex and having an intra oral site were included in the study. All of these patients underwent surgery for curative cure. Necrosed, scanty and autolysed tissue samples were excluded. Slides were prepared from fresh sections (3 to 5 μm) of paraffin blocks using Accu cut rotary microtome (SRM 200 Sakura, Japan) and were stained with haematoxylin and eosin stains. Histological diagnosis was confirmed by a consultant histopathologist and grading was done by Anneroth’s grading system (Akhter et al., 2011) and applied with *Cyclin D1* and *Ki-67* immunohistochemistry. 

*Immunohistochemistry*

We used Monocolonal antibody to Clone 7B11 (Catalogue no: 18-0192Z; invitrogen, USA) for *Ki-67 *at a concentration of 1:100 to 1:200 and for *Cyclin D1 *monoclonal antibody of Vision bio-systems Novocastra Kit was applied.

Immunohistochemistry was performed on paraffin embedded tissue sections of 3-4 µm thickness which were mounted on glass slides with a suitable tissue adhesive. For antigen retrieval, tissue sections were deparaffinised and rehydrated with distilled water. To block any endogenous activity, sections were placed in 3% hydrogen peroxide for 3 minutes and were then washed in running tap water. Antigen retrieval was carried out by heating 1,500 ml of the recommended retrieval solution (0.01 M citrate buffer, pH 6.0) until boiling in a pressure cooker. When the pressure cooker reached operating temperature and pressure, they were left for 1 minute and then removed and placed immediately in cool tap water. The sections were washed in Tris Buffer Saline (TBS) for 1-5 minutes and then diluted in normal serum for 10 minutes. They were incubated overnight at 4^o^C with primary antibody (*Cyclin D1* and* Ki-67*) and washed in TBS for 2-5 minutes and were later incubated in appropriate biotinylated secondary antibody for 60 min at room temperature (mouse EnVision System HRP, Dako Cytomation). In order to visualize, slides were incubated in Diaminobenzidine and then counterstained with haematoxylin, dehydrated and mounted. Mantle cell lymphoma was taken as a positive control for *Cyclin D1* and tonsil tissue for *Ki-67*.

The prepared sections were evaluated by a consultant histopathologist on Olympus B x 60 light microscope and the staining intensity of tumour cells was classified semi-quantitatively. Labelling index was determined by counting stained cells in 10 consecutive microscopic high power fields in each section (original magnification x 400). Percentages of positive stained nuclei were calculated by dividing with total no of cells in the specific area x 100 and categorized into 2 groups; mild positive (0-49%) and strong positive ( ≥50) (de Vicente et al., 2002).

*Statistical analysis*

The data was analyzed using SPSS software (version 19;SSPS Inc., Chicago, IL, USA). Means were calculated for tumour size and age of the patient. Frequency and percentages were calculated for gender, site, grades of the tumour and immunohistochemical expression (*Cyclin D1* and *Ki-67*). Chi square test was applied to observe the difference in expression of these two markers in primary and lymph node metastatic OSCC and the association of *Ki- 67* and *Cyclin D1* with the different intraoral locations of these both entities. A p value of ≤ 0.05 was considered as statistically significant.

## Results

A total of 30 cases, 15 each of primary and metastatic OSCC were included in the study. Primary tumours showed equal number of grade I, II and III whereas in metastatic 11 (73.3%) belonged to grade I, 4 (26.7%) were in grade II while no grade III tumour was found. The age of the patients with primary OSCC ranged from 24 to 80 years having a mean of 53.47 ±16.67, whereas for metastatic tumours it ranged from 40 to 80 years with a mean of 61.47 ±11.94. We noted a male predominance in both the primary and metastatic tumours; 80.8% in primary and 86% with lymph node metastatic tumour. Tongue was the most frequently involved site for both the primary (40%) as well as metastatic (53%) OSCC. The mean tumour size noted was 1.16 ±0.66 and 3.93 ± 0.81 mm for primary and metastatic type respectively.

*Cyclin D1* and* Ki-67* proteins expressions were detected in the nuclei of the cells at various levels. The expression of *Cyclin D1* was found strong positive in 26% (4) of the primary cases, the remaining 11 (74%) were mild positive. In metastatic tumours 20% were mild positive whereas 80 % were strong positive for it showing a significant statistical difference (p value 0.003). For *Ki-67*, expression was seen mild positive in 8 (53.33%) cases of primary OSCC and the rest 7 (46.67%) were strongly positive and a opposite was noted in metastatic tumours; p value was found insignificant here (0.715). It was also found that there is no significant correlation between tumour differentiation (grades) and *Ki-67* and Cyclin D1 expression in both primary and metastatic tumours (p> 0.05). The expression of both of the markers (*Cyclin D1 *and *Ki-67*) in different grades of both types of tumours are shown in Table 1 and 2. 

The involved sites of primary tumour were tongue, floor of the mouth, buccal mucosa, palate and gingiva. On the other hand none of the metastatic tumour was found in palate or gingiva. There was also an insignificant association of *Ki- 67* (p = 0.22) and *Cyclin D1* (p= 0.36) when compared with various involved locations of primary tumours. Similarly expression of *Ki- 67* and *Cyclin D1* in metastastic tumours was also insignificant (p= 0.44 and 0.99 respectively) when compared in the sites of involvement. 

**Table 1:** Expression of Cyclin D1 and Ki-67 in Histological Grades of Primary OSCC

	Cyclin D1		Ki-67	
Grades	Low intensity <50 (Mild Positive) n (%)	High intensity >50 (Strong Positive) n (%)	Low intensity <50 (Mild positive) n (%)	High intensity >50 (Strong positive) n (%)
Grade I	4 (26)	1 (6.6)	3 (20)	2 (13)
Grade II	4 (26)	1 (6.6)	3 (20)	2 (13)
Grade III	3 (20)	2 (13)	2 (13)	3 (20)
Total	11 (73.3)	4 (26.7)	8 (53.3)	7 (46.7)

**Figure 1:**
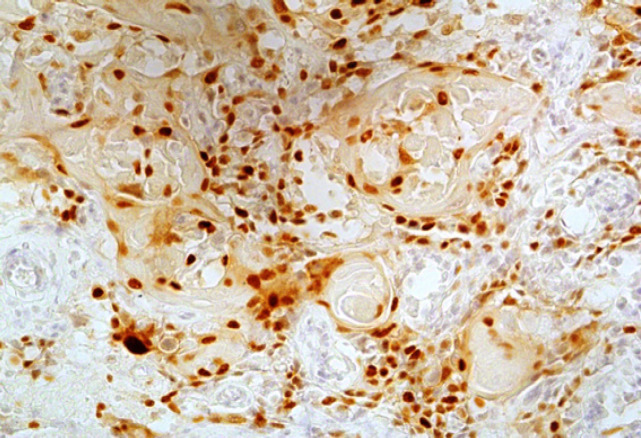
High Intensity (Strong Positive) Expression of Cyclin D1 in Metastatic OSCC (x100).

**Figure 2:**
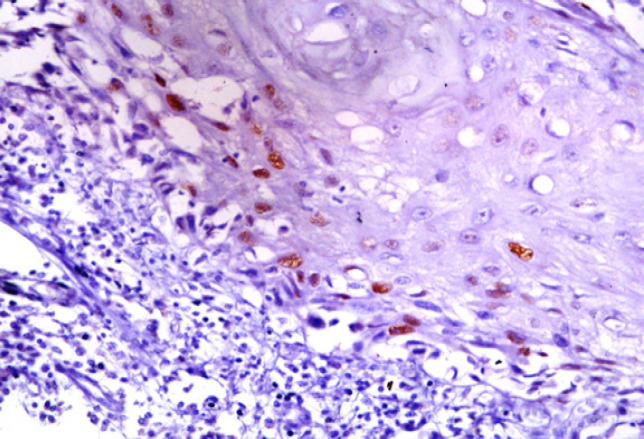
Low Intensity (Mild Positive) Expression of CyclinD1 in Primary OSCC (x100).

**Figure 3:**
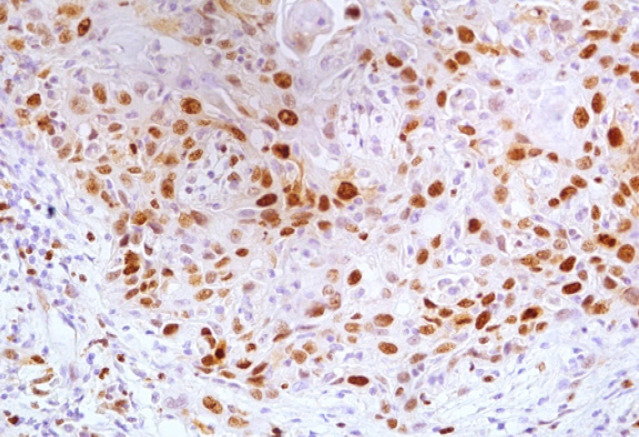
High Intensity (Strong Positive) Expression of Ki-67 in Metastatic OSCC (x100)

**Figure 4:**
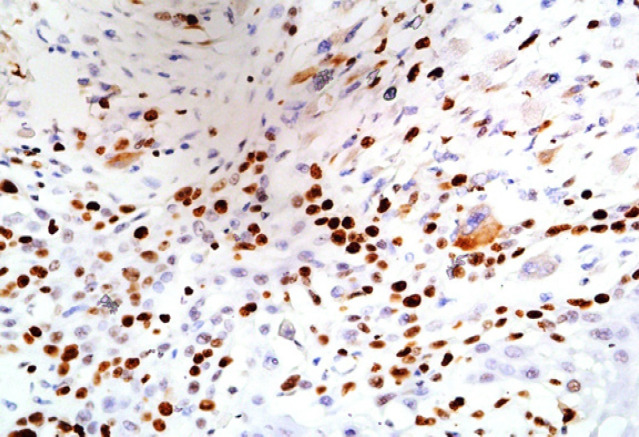
High Intensity (Strong Positive) Expression of Ki-67 in Primary OSCC (x100)

**Table 2:** Expression of Cyclin D1 and Ki-67 in Histological Grades of Metastatic OSCC

Grades	Cyclin D1		Ki-67	
	Low intensity <50 (Mild Positive) n (%)	High intensity >50 (Strong Positive) n (%)	Low intensity <50 (Mild positive) n (%)	High intensity >50 (Strong positive) n (%)
Grade I	1 (6.6)	10 (66)	5 (33)	6 (40)
Grade II	2 (13)	2 (13)	2 (13)	2 (13)
Total	3 (20)	12 (80)	7 (46.6)	8 (53)

## Discussion

As evident from previous studies results, oral squamous cell carcinoma (OSCC) comprises 80 to 90% of all malignant neoplasm and is the most common oral malignancy. Although incidence of oral cancers is highly inconsistent worldwide, still it is an established fact that oral cavity is 6^th ^to 9^th^ most common anatomical location for cancer, depending on the specific region in some countries and gender of the patients, especially Southeast Asia is the most familiar representing locality for patients of OSCC. (Pires et al., 2013).

*Ki-67* and Cyclin D proteins are the two main biomarkers used to illuminate the clinical behaviour of OSCC.* Cyclin D* is a cell cycle regulator that binds to cyclin-dependent kinase *(CDK) 4* and* CDK6*. This *cyclin-CDK* complex plays a key role on the induction of DNA synthesis and transition of cells between G1/S phases making it a true target during carcinogenesis. An elevated *Cyclin D1* expression has been associated with lymph node involvement and poor prognosis (Guimarães et al., 2015).

We also noticed a similar trend; 4/15 primary and 12/15 metastatic tumours showed strong positivity for it (Figure 1 and 2) and the results were statistically significant as well. The higher expression of *Cyclin D1 *in metastatic tumours can be explained by the fact that alterations are related to strong proliferative activity and invasive mode of inclination of the lesions. Likewise metastasis of OSCC in lymph node is an important factor in clinical outcome of patients. Shiang-Fu et al., (2012) mentioned this fact of lymph node metastasis as the major determinant of OSCC outcomes and over expression of *Cyclin D1* was significantly associated with lymph node metastasis.

Similarly Capaccio et al., (2000) mentioned the lack of existence of accuracy in the clinical evaluation of cervical metastasis and emphasized a need to enhance the affectivity of the preoperative evaluation of neck lymph nodes with the aim to reduce the rate of false negatives. They basically recommended the use of the immunohistochemical analysis of *Cyclin D1* expression in diagnostic biopsy samples for patients prior to treatment with elective neck dissection. Contrarily, Oshini et al., (2014) did not predict any significant difference of its expression in primary and metastatic foci of OSCC. 

The expression level did not correlate with tumour differentiation/ grades. Analysis of *Cyclin D1* expression in primary OSCC did not reveal any difference among the three histological grades of tumours. High intensity (strong positivity) was seen in only one case each of grade I and II tumours and 2 cases of grade III tumours. A similar trend was noted in metastatic tumours which too had no difference of expression in grade I and grade II tumours. A Brazilian study by Neves et al., (2004) showed positivity for *Cyclin D1* in 24 of 28 (85.7%) OSCC cases. They demonstrated an increased expression in high grade tumours which is not comparable to our study. However, we could not retrieve any grade III metastatic tumour which can be resulted due to limited sample size or the participants were diagnosed at early stages of their metastasis.

*Ki-67* nuclear antigen, a cell proliferative marker also known as MKI67 has provided a reliable method to estimate tumour intensification rate. It is a basic protein expressed in all active phases of the cell cycle (S, G2, and mitosis) and shows increase expression with the cell progression (Nidhi et al., 2013). We found its high intensity of expression in 7 of 15 primary and 8 of 15 metastatic tumours in our study. The staining intensity was also non-comparable between the two (Figure 3 and 4). We used monoclonal antibody for *Ki-67* as the studies indicated its validity in higher immunoreactivity (Lindboe and Torp, 2002). On the other hand Oshini et al., (2014) reported that *Ki 67* expression was significantly higher in metastatic foci than in Primary foci. 

We had a limited number of surgically treated patients with dissection particularly having cervical lymph node metastasis which can be a limitation. Our sample size was limited to 30 due to the fact that the specimen containing primary OSCC with a positive metastatic lymph nodes were rarely available. Further research on larger sample size is required to elucidate the role of these markers in differentiating primary and metastatic OSCC on small incisional biopsies.

In our country, this is the first study to be conducted at AFIP. Both of these markers showed statistically insignificant expression in different histological grades of primary and metastatic OSCCs. *Cyclin D1 *however, demonstrated marked difference of expression in primary and metastatic OSCC regardless of their grades. Thus it can be a useful tool to predict metastatic/ aggressive behaviour of a tumour on small biopsies. No doubt, follow up of such patients having strong *Cyclin D1* positivity (high intensity) on premier biopsies is essential for better prediction of their prognosis/ outcomes.
